# Correlation Analysis of the Carboxyl and Carbonyl Groups of Natural Organic Matter and the Formation Potential of Trihalomethanes and Chloral Hydrate

**DOI:** 10.3390/molecules27217454

**Published:** 2022-11-02

**Authors:** Xinwei Zhu, Minghua Li, Pengwei Yan, Jimin Shen, Jing Kang, Zhonglin Chen

**Affiliations:** State Key Laboratory of Urban Water Resource and Environment, School of Environment, Harbin Institute of Technology, Harbin 150090, China

**Keywords:** disinfection by-products, dissolved organic matter, functional groups, pre-oxidation

## Abstract

Natural organic matter (NOM) has always been considered the main precursor of disinfection by-products (DBPs) during the chlorine disinfection of drinking water. This research focuses on investigating the correlation between the functional group (carboxyl and carbonyl groups) content of NOM and the formation of trichloromethane (TCM) and chloral hydrate (CH). The quantitative determination of carboxyl groups, carbonyl groups, TCM, and CH were conducted during the drinking water treatment processes with different coagulant dosages and with/without pre-oxidation by KMnO_4_ or NaClO. The most appropriate coagulant for the removal of conventional components was polyaluminum chloride (PAC), and the dosage was 110 mg/L. Up to 43.7% and 14.5% of the carboxyl and carbonyl groups, respectively, were removed through the coagulation and sedimentation processes, which can be enhanced by increasing PAC dosage. The filtration process further increased the removal rates of these two functional groups to 59.8% and 33.5%, respectively. The formation potential of the TCM and CH decreased as the PAC dosage increased. Pre-oxidation by KMnO_4_ (0.8–1.0 mg/L) effectively controlled the formation of DBPs while increasing the carboxyl and carbonyl group content. Pre-oxidation by NaClO decreased the formation of TCM rather than CH, and a suitable amount (0.5–1.0 mg/L) decreased the carboxyl and carbonyl groups. It was found that there was a good linear correlation between carboxyl groups and TCM and CH. The linear fit R^2^ values of the carboxyl groups to TCM and CH were 0.6644 and 0.7957, respectively. The linear fit R^2^ values of the carbonyl groups to TCM and CH were 0.5373 and 0.7595, respectively.

## 1. Introduction

Chlorine disinfection, which can effectively kill pathogenic bacteria and microorganisms to ensure water quality safety, is widely used in the water treatment process [1]. However, contact between the disinfectant and the ubiquitous organic matter in water causes the formation of disinfection by-products (DBPs), such as trihalomethanes (THMs), halogenated aldehydes (HALs), etc. More than 700 kinds of DBPs have been disclosed in the past few decades, and these present a great threat to human health [2]. THMs may increase the risk of bladder and colon cancer [3]. Trichloromethane (TCM) was the most widely detected [4]. HALs are ubiquitous and have been reported to be the third largest group of DBPs by weight [5]. Chloraldehyde hydrate (CH) is the most abundant individual HAL species, and it has been shown to be more genotoxic than TCM [6]. As a result, restrictive regulations on DBP concentration have been enacted around the world. For example, in China, the acceptable concentration of TCM has been limited to 60 μg/L, and, in addition, the sum value of THMs has been limited to 1 mg/L. The threshold of THMs is 80 μg/L according to the EPA of the USA. CH is limited to 10 μg/L in China. Other countries also have threshold requirements for CH. It is meaningful and necessary to study the formation and transformation rules of these DBPs for their control.

The background of natural water quality is complex; it typically contains some metal ions and inorganic and organic matter [7,8]. Various technologies have been applied to remove these contaminants, such as adsorption, oxidation, and filtration [9,10]. However, natural organic matter (NOM), consisting of organic matter with different molecule weights (MWs) and hydrophobicity, cannot be totally degraded through physical or chemical means, thus making it the main precursor of DBP formation [11,12]. Removing NOM from water is an effective means to control the formation of DBPs [13]. The characteristics of NOM greatly affect the type and concentration of DBP that can form. For example, some researchers found that NOM with a low MW was more likely to form THMs and CH [14]. The hydrophobic parts of NOM were more likely to form chloropicrins and dichloroacetonitrile. Therefore, revealing the relationship between the structural characteristics of NOM and the formation of DBPs is of great significance for the proposal of control strategies. NOM is composed of a class of aromatic rings with different oxygen-containing functional groups, which are the chemical reaction centers in the chlorination process. For example, phenols (Ar-OH) have been proven to play the main role in the formation of CH [15]. However, there are few studies focused on the correlation between the functional groups of NOM and DBP formation.

The variation in the amount and the structural characteristics of the NOM observed during the water treatment process aroused much attention and revealed the DBP formation mechanism. In practice, the removal of the DBPs’ precursor in the drinking water treatment process mainly depends on the conventional treatment methods, including coagulation, sedimentation, and filtration. The removal efficiency was greatly affected by the molecular weight and hydrophilicity of the NOM. For example, the hydrophilic parts of the NOM with low MWs were hard to remove by coagulation using FeCl_3_ or polyaluminum chloride (PAC) as coagulants [16], while the hydrophobic parts with high MWs were easier to remove by coagulation or adsorption [17]. The coagulant dosage was an important factor in the daily operation of the drinking water treatment plant. Insufficient coagulant would cause poor coagulation, while an excessive dosage would lead to poor floc settling performance, resulting in poor NOM removal. However, there were few studies focused on the influence of coagulant dosage on NOM characteristics and DBP formation potential in the effluent of the sedimentation and filtration.

Only partial NOM can be removed by the conventional water treatment process, thus resulting in different DBP formation patterns. Pre-oxidation is usually applied to intensify the coagulation process. The pre-oxidation process can remove part of the organic matter in water, thereby changing the structural characteristics of the NOM [18,19]. KMnO_4_ is a conventional chemical for pre-oxidation that is effective in removing trace organic contaminants and TCM precursors [20]. Chen et al. reported that pre-oxidation by KMnO_4_ intensified coagulation, increasing the algae removal rate by 10–30% [21]. NaClO is another conventional chemical for pre-oxidation, though it is also used as a disinfectant. NaClO can cause lysis of the inner cell and is much stronger than KMnO_4_ [22]. Moreover, the variation of organic matter by the pre-oxidation process causes different DBP formation characteristics, which needs further investigation.

In this study, source water with high NOM content was used as the research object. The traditional water treatment process was simulated in the laboratory, and the variation of NOM characteristics and DBP formation were investigated. First, the effect of the coagulant dosage on NOM removal and DBP formation after sedimentation and filtration during the conventional treatment process was studied. Second, the influence of pre-oxidation using KMnO_4_ and NaClO was studied. Third, the quantitative determination of carboxyl and carbonyl groups during the treatment process was conducted. Last, the TCM and CH formation potential and their relationship with carboxyl and carbonyl groups were analyzed. The molecular structure of the NOM was divided into functional groups so as to study the relationship between the molecular structure and the formation of DBPs, which will help to reveal the mechanism of DBP formation and the structural characteristics of NOM.

## 2. Materials and Methods

### 2.1. Chemicals and Reagents

Commercial sodium hypochlorite (NaOCl, 4.00–4.99%, as Cl_2_), KMnO_4_ (≥99.0%), and CH (≥99.0%) were purchased from Merck (Darmstadt, Germany). A stock solution of NaOCl was prepared by diluting the commercial solution into 800 mg/L (as Cl_2_), which was calibrated using Na_2_S_2_O_3_. A four regulated THM (THM_4_) mix standard solution (1000 μg/L), including TCM, bromodiodomethane (CHBrCl_2_), dibromochloromethane (CHBr_2_Cl), and bromoform (CHBr_3_), was purchased from o2si^@^ smart solution. Milli-Q water (18.2 Ω/cm) was used to prepare the solutions.

A raw water sample (RS) was collected from the inlet of a water treatment plant, which is located in northeast China and uses reservoir surface water as its source. Water samples were transported to the laboratory within 2 h and then stored at 4 °C before being analyzed. The properties of the RS are shown in Table 1.

### 2.2. Experimental Procedure

A small-scale water treatment system (including coagulation, sedimentation, and filtration processes) was established in the laboratory to simulate the conventional water treatment process. Briefly, a six-way agitator was used as the coagulation and sedimentation reactor. The process of coagulation was operated according to a pre-programmed procedure (Table S1, supporting information, SI) after adding a specific amount of PAC, and then 30 min was needed for sedimentation. Filtration was performed in a prepared column filled with sand. Samples were collected after sedimentation and after filtration, labeled as S1 and S2, respectively. The pre-determined amounts of NaClO and KMnO_4_ was added before coagulation and stirred for 2 min to start the pre-oxidation process.

In this study, NaClO was used as the disinfectant. The stock solution was prepared by diluting the commercial one into 800 mg/L. Forty milliliters of the water sample was put into a brown vial, and then the pre-determined volume of stock NaClO solution was added to ensure the residual chlorine concentration achieved 1.0 ± 0.2 mg/L after a 24 h-reaction. Na_2_S_2_O_3_ was used to quench the residual chlorine before determining the formation of DBPs. In addition, when NaClO was used as the pre-oxidant, the sum of the amount of NaClO in the pre-oxidation stage and the disinfection stage was kept constant to avoid the interference of excessive disinfectant resulting in the elevation of DBPs.

### 2.3. Analytical Methods

DOC concentration was measured using a Shimadzu TOC/TN analyzer, and the detection limit was 0.1 mgC/L. The permanganate index (PI) was measured by titration with sodium oxalate under acidic conditions. UV_254_ was measured using a spectrophotometer (Beijing Purkinje General T6, Beijing, China) set to 254 nm. The chromaticity was measured by Platinum Cobalt Standard Colorimetry. The NaClO concentration (free Cl_2_) was measured by DPD/FAS titration [23].

MTBE was used to extract the DBPs from the water sample. Following this, the volatile DBPs, including TCM and CH, were measured by gas chromatography (GC) with an electron capture detector (ECD) and an HP-5 capillary column (30 m × 0.25 mm i.d., 0.25 μm film thickness, J&W, New Brighton, MN, USA). Standard solutions of TCM and CH were diluted into a series of solutions with a gradient concentration. The concentration of TCM and CH in the samples was calculated through the standard curve. The methods for determining the carboxyl and carbonyl groups are shown in Text S1 (SI).

## 3. Results and Discussion

### 3.1. Removal of NOM by Different Water Treatment Units

**Figure 1:**
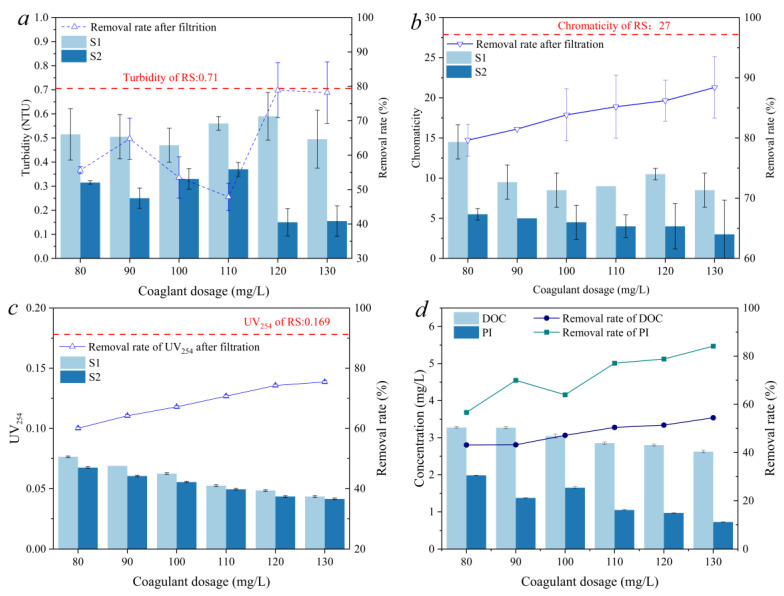
The effect of coagulant dosage on the removal of turbidity (**a**), chromaticity (**b**), UV254 (**c**), and DOC and PI (**d**) in the effluent of sedimentation and filtration units.

The removal of the NOM in the effluent of sedimentation and filtration under different PAC dosages was studied first. As shown in Figure 1a, the turbidity of S1 was around 0.5, which was slightly less compared with the RS (0.71), while the turbidity was effectively removed after filtration, and the removal rate increased from 55.6% to 78.2% as the PAC dosage increased from 80 to 130 mg/L. Compared with the chromaticity of the RS (27), the coagulation and sedimentation processes significantly decreased that of S1 to around 10 when the PAC dosage exceeded 90 mg/L (Figure 1b). The removal of chromaticity was further enhanced after the filtration process, especially under a higher PAC dosage. Compared with the RS, the removal rate of chromaticity in S2 increased from 79.6% to 88.4% as PAC dosage increased from 80 mg/L to 130 mg/L. As Figure 1c shows, the UV_254_ of the RS was 0.169, indicating that a large number of aromatic DOM existed in the raw water [24]. The UV_254_ levels of S1 and S2 were both at a low level (<0.08) and showed a linear decreasing trend with the increase in PAC dosage. However, the improvement of UV_254_ removal by the filtration process was limited. This phenomenon indicates that the aromatic DOM could be efficiently removed by coagulation and sedimentation when PAC is the coagulant, rather than by filtration.

As Figure 1d shows, the removal rate of the DOC and PI both increased as the PAC dosage increased. The removal rates of the PI and DOC increased by 27.5% and 11.3% as the PAC dosage increased from 80 mg/L to 130 mg/L, respectively. DOC and PI are both important water quality parameters that can reflect the amount of DOM and can also be used evaluate the effect of the water treatment process [25]. The DOM removal efficiency increased as the PAC dosage increased; however, this increase was limited. DBPs formed as the residual DOM came into contact with the disinfectants. How the coagulation–sedimentation and filtration processes influenced the formation of DBPs as the dosage of coagulant increases remains a question. It is also important to characterize the properties of DOM because it was the main precursor of DBPs in this research.

It has been speculated that the conventional water treatment process could just remove a part of the DOM [26,27,28]. Some previous research has tried to figure out the variation or transformation of DOM during the water treatment process. Fluorescence spectroscopy combined with parallel factor analysis (PARAFAC) has been applied to analyze portions of DOM [23,28,29]. According to Maqbool’s research, protein-like fluorescence was removed during physical treatment processes such as coagulation–precipitation and sand filtration, while the chemical pretreatment and disinfection unit showed a higher tendency to remove the humic-like fluorescence [28]. The variations of the DOM influence the formation of DBPs [24].

### 3.2. Removal of Natural Organic Matter by the Pre-Oxidation Process

**Figure 2:**
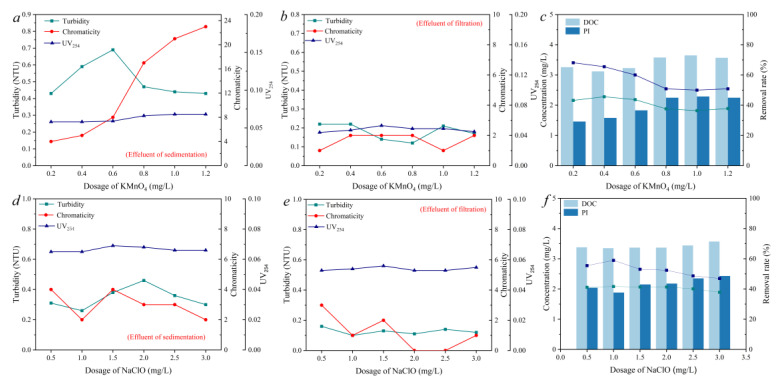
The effect of KMnO_4_ dosage (**a**–**c**) and NaClO dosage (**d**–**f**) on the removal of turbidity, chromaticity, UV_254_, and DOC and PI in the effluent of sedimentation and filtration units.

Considering the limited effect of conventional water treatment processes on the removal of NOM in raw water, the pre-oxidation process using KMnO_4_ and NaClO was applied to intensify the PAC coagulation process. As is shown in Figure 2a, with the increase in the KMnO_4_ dosage from 0.2 mg/L to 1.2 mg/L, the UV_254_ content of S1 was effectively reduced to around 0.06 from the 0.169 of the RS, though the turbidity and chromaticity were still at a high level. At the same time, it was observed that when the KMnO_4_ dosage increased, the color of the water sample gradually turned brown, which was attributed to the precipitation of the manganese dioxide produced in the oxidation process. As Figure 2b shows, the turbidity, chromaticity, and UV_254_ of S2 were further reduced. However, the increase in KMnO_4_ did not increase the removal of DOC and PI compared with the control group. In addition, the concentration of the DOC and PI in S2 increased as the KMnO_4_ increased from 0.2 to 1.2 mg/L. The above results indicate that the KMnO_4_ pre-oxidation process facilitated the effective removal of suspended matter by sand filtration Partial aromatic organic matter could also be removed. However, the decomposition of macromolecular organic matter into small molecular species during pre-oxidation may lead to elevated DOC and PI concentrations in treated water.

Pre-oxidation using NaClO was conducted to compare its effect with that of KMnO4. Different dosages (0.5–3.0 mg/L) of NaClO were applied to evaluate its role in intensifying the coagulation process. As Figure 2d shows, when the dosage of NaClO was 1.0 mg/L, the turbidity and chromaticity of S1 were 0.26 NTU and 2, respectively. Moreover, the turbidity and chromaticity of S2 after filtration were 0.1 NTU and 1, respectively, which were reduced significantly compared with the control group without the pre-oxidation stage. While there was a slight difference in turbidity and chromaticity removal using the same dosage of NaClO and KMnO_4_, the UV_254_ of S2 was 0.053 when 1.0 mg/L of NaClO was added, which was a little higher compared with that of KMnO_4_ at same dosage (0.049), indicating the insufficient removal of aromatic DOM. Furthermore, different NaClO dosages had no obvious effect on the UV_254_ of S1 and S2, which remained in the range of 0.053–0.069 (Figure 2e). When the NaClO dosage was 1.0 mg/L, the removal rates of DOC and PI after filtration were 41.6% and 59.0%, respectively, both of which were enhanced significantly compared with those obtained using the same dosage of KMnO_4_. However, with the increase in the NaClO dosage, both the DOC and PI of S2 increased noticeably. When the NaClO dosage increased from 0.5 mg/L to 3.0 mg/L, the removal rates of the DOC and PI decreased from 41.7% and 55.5% to 37.8% and 46.9%, respectively (Figure 2f). NaClO may destroy the cellular structure of microorganisms in water and release organic matter, thus increasing the content of organic matter in water.

Although the intensification of the pre-oxidation process by the coagulation process has been regarded as an effective means to control the formation of DBPs in previous studies, it was necessary to pay attention to the variation of organic matter when there was substantial NOM contained in the raw water. Natural macromolecular organics may be decomposed into small molecular organics during the pre-oxidation process, and these small molecular organics were difficult to removed effectively during the subsequent coagulation, precipitation, and filtration processes, so there was a risk of generating DBPs during the disinfection process.

### 3.3. Quantitative Analysis of Organic Functional Group Content in the Effluent of Different Treatment Units

**Figure 3:**
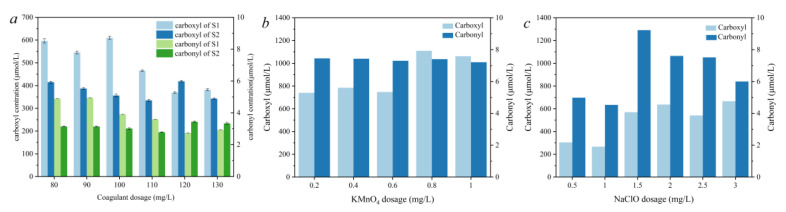
Quantitative determination of carboxyl and carbonyl groups in the effluent of sedimentation and filtration under conventional treatment (**a**) and KMnO4 (**b**) or NaClO (**c**) pre-oxidation.

The amount of carboxyl and carbonyl groups in the RS was 824 μmol/L and 4.21 μmol/L, respectively, indicating that the components of DOM were mainly composed of carboxyl groups. According to the 3D-EEM analysis of the same water source from our previous research, the DOM of the raw water was mainly composed of humic acid and fulvic acid [23]. Here, the variation of the amount of the two functional groups during the water treatment process was evaluated when a different dosage of PAC was applied in the coagulation process. As Figure 3a shows, the carboxyl and carbonyl groups of S1 both decreased as the PAC dosage increased. When the PAC dosage was 110 mg/L, the content of the carboxyl groups in S1 was 463.5 μmol/L, and the removal rate was 43.7%. When the PAC dosage was increased to 120 mg/L and to 130 mg/L, the carboxyl content was 367.16 μmol/L and 384.6 μmol/L, respectively, and the removal rate remained at about 55%, indicating that the removal effect of the coagulation process on carboxyl organic compounds is limited. The carboxyl groups can ionize acetate ions and be negatively charged, while aluminum coagulants will be positively charged after hydrolysis. The removal effect of coagulation from precipitation to the carboxyl groups may be the result of charge neutralization. When the PAC dosage was 110 mg/L, the carbonyl content of S1 was 3.6 μmol/L, and the removal rate was 14.5%. After increasing the dosage of PAC to 120 mg/L and to 130 mg/L, the carbonyl content of S1 was 2.72 μmol/L and 2.91 μmol/L, respectively. When the PAC dosage was 120 mg/L, the rate of removal of carbonyl compounds by coagulation and sedimentation reached its maximum, and further increasing the dosage of coagulant made no obvious improvement. The coagulants could interact with the DOM in different ways and could remove some organic matter through precipitation [16,30].

The carboxyl and carbonyl group content of S2 changed slightly as the PAC dosage increased from 80 mg/L to 130 mg/L, indicating that the coagulation and sedimentation process has little influence on the removal of organic matter containing these two functional groups in the filtration process. Nevertheless, the filtration process could further enhance the removal of carboxyl and carbonyl groups. For example, the carboxyl group content of S2 decreased to 331.5 μmol/L and to 463.5 μmol/L in S1 when the PAC dosage was 110 mg/L.

The change in the carboxyl and carbonyl group content of S2 was also evaluated when KMnO_4_ pre-oxidation was conducted. The PAC dosage was set at 110 mg/L. As is shown in Figure 3b, the pre-oxidation of KMnO_4_ significantly increased the carboxyl and carbonyl group content in S2. When the KMnO_4_ dosage was 0.2 mg/L, the carboxyl group content was 740.4 μmol/L, which was obviously higher than that of control group (463.5 μmol/L). Moreover, the carboxyl content increased to 1061.7 μmol/L as the KMnO_4_ dosage increased to 1.0 mg/L, which was significantly higher than that of the RS (824 μmol/L). Similarly, the carbonyl group content increased compared with the control group without KMnO_4_ pre-oxidation. However, the dosage of KMnO_4_ had little effect on the carbonyl content, which was around 7.3 μmol/L. This phenomenon indicates that even though pre-oxidation by KMnO_4_ enhanced the removal of turbidity and chromaticity, the characteristics of the DOM were changed. Because of the oxidation capacity of KMnO_4_, cells of aquatic plants such as algae or microorganisms in the water were ruptured, thereby releasing organic matter, which can also be seen from the increased DOC content in the measured water sample.

When NaClO was used as the pre-oxidant, both the carboxyl and carbonyl group content of S2 were lower compared with those under KMnO_4_ pre-oxidation. From Figure 3c, the carboxyl group content was 266.8 μmol/L when the NaClO dosage was 1.0 mg/L, which was significantly lower than that of the control group without the pre-oxidation stage (463.5 μmol/L). However, the carboxyl group content increased to 666.7 μmol/L as the NaClO dosage increased to 3.0 mg/L. The carbonyl content increased from 5.0 μmol/L to 9.2 μmol/L as the NaClO dosage increased from 0.5 mg/L to 1.5 mg/L. Nevertheless, continuing to increase the NaClO dosage to 3 mg/L resulted in a decrease in the carbonyl content to 6.0 μmol/L. This may be because small amounts of NaClO cannot destroy algae and microbial cells in water and it can only partially degrade NOM. If the NaClO dosage increased, the released organic matter could also be degraded, thus resulting in a decrease in carbonyl group content.

### 3.4. Generation Potential of Disinfection by-Products in Different Treatment Units

**Figure 4:**
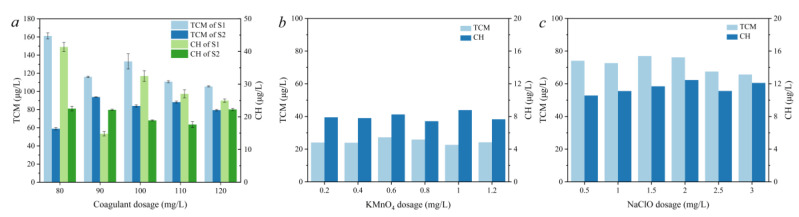
Quantitative determination of TCM and CH in the effluent of sedimentation and filtration under conventional treatment (**a**) and KMnO4 (**b**) or NaClO (**c**) pre-oxidation.

Both the S1 and S2 water samples obtained under different conditions were chlorinated for 24 h to evaluate the concentration of the generated DBPs. As Figure 4a shows, the formation of TCM was much higher than that of CH in all conditions. Both the formation of TCM and CH in S1 and S2 decreased as the PAC dosage increased, indicating that the coagulation process could efficiently remove the precursor of TCM and CH. However, both the TCM and CH concentrations of S1 were relatively high compared with acceptable levels. When the PAC dosage was 120 mg/L, the TCM and CH concentrations of S1 were 105.1 μg/L and 24.6 μg/L, respectively, indicating the limiting effect of the coagulation and sedimentation processes on the control of DBPs. Nevertheless, the filtration process significantly enhanced the removal of the precursor of TCM. When the PAC dosage was 80 mg/L, the TCM concentration of S2 decreased from 161.2 μg/L (S1) to 59.8 μg /L, indicating the important role of the filtration process in controlling TCM formation. Similarly, the CH concentration of S2 decreased from 41.4 μg/L (S1) to 22.0 μg/L. However, with the increase in PAC dosage, the enhancement of the filtration gradually decreased. Excessive coagulant would lead to loose flocs and would not be conducive to sedimentation, thus affecting the removal effect of organic matter. Moreover, the TCM and CH concentrations of S2 were both at a high level that exceeded the acceptable concentration according to Chinese law. Therefore, the effect of pre-oxidation on the control of DBPs was further studied.

As Figure 4b shows, compared with the control group without the pre-oxidation intensified conventional water treatment process, the formation of TCM and CH was significantly reduced by KMnO_4_ pre-oxidation. When the dosage of KMnO_4_ was 0.8 mg/L, the amount of CH was 7.42 μg/L; when the dosage of KMnO_4_ was 1.0 mg/L, the formation of TCM was 22.62 μg/L. This phenomenon indicates that the KMnO_4_ pre-oxidation process can effectively reduce TCM and CH formation in the filtrated water, and the appropriate dosage of KMnO4 should be in the range of 0.8–1.0 mg/L.

When NaClO was used as a pre-oxidant, to ensure the consistency of the chlorine dosage during the disinfection experiments, the total chlorine (including that used in pre-chlorination and post-chlorination) was controlled at 3.5 mg/L. As is shown in Figure 4c, with the increase in the dosage of NaClO, the formation of TCM exhibited a downward trend, while the formation of CH increased. When the NaClO dosage was 2.0 mg/L, the formation of the CH was highest. However, the formation of the TCM was well controlled when more NaClO was added in the pre-oxidation period. Thus, pre-oxidation using NaClO can effectively control the formation of TCM, but it cannot effectively control the formation of CH, and may even lead to its increase.

The formation of DBPs was determined by varieties and the quantity of DOM [8,31]. DOM from different source and with different components can affect DBP formation under the same conditions [1,24,32,33]. In our previous research [23], the formation of THM and CH was strongly related to the amount of humic acid in the five components according to the PARAFAC model. In another study [13], MWs of 0–1 kDa and 5–10 kDa, as well as hydrophobic DOM, determined the formation of DBPs in raw water, and hydrophobic fractions could not be efficiently removed by the conventional water treatment process, contributing to the DBP precursors.

### 3.5. Correlation Analysis between Functional Group Content and Generation Potential of Disinfection by-Products

An in-depth understanding of the relationship between the properties of NOM in water and the formation of DBPs is necessary to reveal the mechanism of DBP formation and propose effective control strategies [25,34]. Here, the relationships between TCM and CH formation and carboxyl and carbonyl groups are evaluated. All the data obtained in the water treatment process were used to analyze the correlation.

To explore the correlation between the organic functional groups in water and the formation potential of disinfection by-products, this section linearly fits the above-mentioned determined carboxyl and carbonyl content and the DBP formation potential. A linearity of the functional carboxyl groups and carbonyl content measured in the same water sample (including post-emergence water and filtered water) with the maximum disinfection by-product formation potential was observed, and a linear correlation between the two functional groups and the three disinfection by-products was observed.

**Figure 5:**
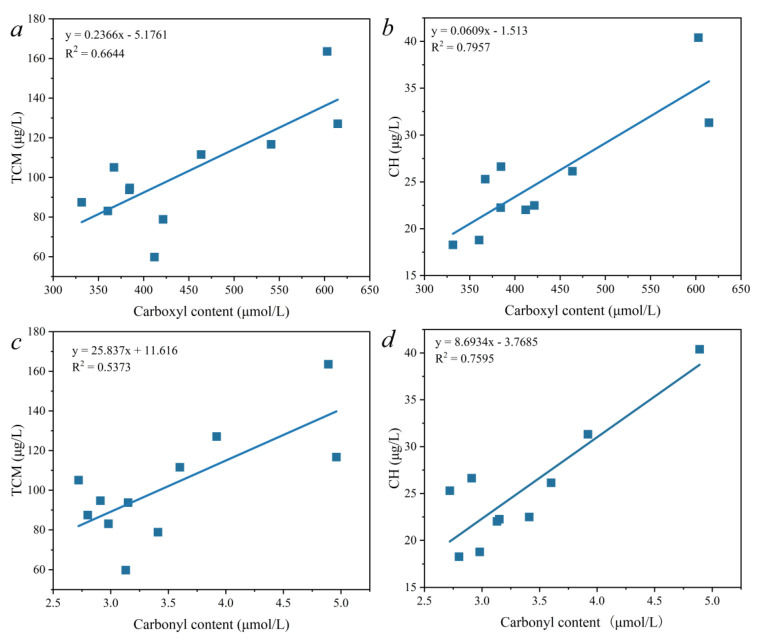
The relationship between DBPs and functional groups. Carboxyl and TCM (**a**), carboxyl and CH (**b**), carbonyl and TCM (**c**), and carbonyl and TCM (**d**).

The fitting results of the carboxyl groups and the THM and CH production amount are shown in Figure 5a,b. It can be seen from the results shown in the figure that the linear fitting R^2^ values of the carboxyl groups and the TCM and CH were 0.6644 and 0.7957, respectively. The carboxyl groups had the strongest correlation with CH, followed by TCM. Figure 5c,d show the linear correlation analysis between TCM and carbonyl content and between CH and carbonyl content, respectively. The linear fitting R^2^ values of carbonyl and TCM and carbonyl and CH were 0.5373 and 0.7595, respectively. The linear correlation between carbonyl and CH was the strongest, followed by that of carbonyl and TCM.

In summary, the formation of CH and TCM is most strongly correlated with the carboxyl group and carbonyl group content of oxygen-containing functional groups, while hetero atomic disinfection by-products containing other halogenated elements do not have a strong linear correlation with oxygen-containing functional groups. The main reason is that the amount of such disinfection by-products is affected by the content of the halogen element. The formation mechanism of such disinfection by-products therefore needs further research.

## 4. Conclusions

The variation of NOM and DBPs formation potential in the conventional drinking water treatment process with and without the pre-oxidation stage was evaluated. The most appropriate PAC dosage (110 mg/L) was determined by considering the conventional components, including DOC, PI, turbidity, chromaticity, and UV_254_. With an increase in the PAC dosage, the concentration of carboxyl groups and carbonyl groups in the water after precipitation showed an obvious downward trend. The filtration process enhanced the removal of the two functional groups, though this was not influenced by PAC dosage. The carboxyl group content after filtration decreased to 331.5 μmol/L, a 59.8% reduction compared with raw water (824 μmol/L). The carbonyl group content after filtration decreased to 2.8 μmol/L, a 33.5% reduction. The KMnO_4_ pre-oxidation stage caused the carboxyl and carbonyl group content after filtration to be higher than that of the source water. The dosage of KMnO_4_ had little effect on the carbonyl content. When the NaClO dosage was in the range of 0.5–1.0 mg/L, the carboxyl and carbonyl content was lower than that of the source water, while a further increase in the NaClO dosage as pre-oxidant caused an increase in the two functional groups. The TCM and CH formation potential after coagulation and sedimentation decreased as the PAC dosage increased. Filtration further decreased the formation of TCM and CH. A good linear correlation between the carboxyl groups and the TCM and CH was observed: the linear fitting R^2^ values of the carboxyl groups and the TCM and CH were 0.6644 and 0.7957, respectively. The linear fitting R^2^ values of the carbonyl groups and TCM and CH were 0.5373 and 0.7595, respectively. The formation of TCM and CH was more dependent upon the carboxyl group content of the NOM. This study has provided a new perspective for understanding the molecule structure of NOM and the formation mechanisms of TCM and CH. Nevertheless, more studies should be conducted to investigate the functional groups of NOM because of their complexity and diversity. Revealing the relationships between the formation of DBPs and the structural features of NOM will provide new insights into the mechanisms responsible for the formation of DBPs as well as strategies for their control.

## Figures and Tables

**Table 1:** Source water quality parameters.

Water Index	Value
Temperature (°C)	4.0–5.5
Turbidity (NTU)	0.62–0.89
Chromaticity	20–25
pH	6.91–7.18
DOC (mg/L)	5.20–5.74
UV_254_ (Abs)	0.167–0.177
PI (mg/L)	4.58–4.8
NH_3_-N (mg/L)	0.15–0.194

## Data Availability

Not applicable.

## Supplementary Materials

The following supporting information can be downloaded at: https://www.mdpi.com/article/10.3390/molecules27217454/s1, Table S1: Procedure for the coagulation process; Text S1: The determination method of carboxyl and carbonyl groups.

## Sample Availability

Samples of the compounds are not available from the authors.
