# Supplementary material for: Correlation Analysis of the Carboxyl and Carbonyl Groups of Natural Organic Matter and the Formation Potential of Trihalomethanes and Chloral Hydrate
Source: Molecules. 2022 Nov 2;27(21):7454. doi: 10.3390/molecules27217454 (PMC9653842; doi:10.3390/molecules27217454)
Supplement: Supplementary file 1 [file molecules-27-07454-s001.zip › molecules-1947269-SI.pdf]

Supporting Information

Correlation analysis of the carboxyl and carbonyl groups of natural organic matter and the trihalomethanes and chloral hydrate formation potential

Xinwei Zhu, Minghua Li, Pengwei Yan, Jimin Shen*, Jing Kang, and Zhonglin Chen*

State Key Laboratory of Urban Water Resource and Environment, School of Environment, Harbin Institute of Technology, Harbin, 150090, PR China

*Correspondence: shenjimin@hit.edu.cn (J. Shen); Tel: +86-0451-86283028;

zhonglinchen@hit.edu.cn (Z. Chen); Tel: +86-0451-86287000;

Table S1 Procedure for the coagulation process

Stage	1	2	3	4
Rotate speed (r/min)	300	60	40	25
Time (min)	1	5	5	5

Text S1 The determination method of carboxyl and carbonyl groups

The method of determining carboxyl and carbonyl groups was exploited according to the Boehm titration. Here, the procedure of the method was proposed in detail. According to Schnitzer, calcium acetate ion exchange method was proper to determinate amount of carboxyl group of humic acid or fulvic acid. The principle of this method is the carboxyl group of organic matter would exchange ion with calcium acetate to generate acetic acid. And then NaOH is added into the solution containing acetic acid to regulate the pH into 9.8. Based on the amount of NaOH consumed by the blank and the sample, the amount of carboxyl group of the sample could be calculated. In order to ensure the accuracy of this method, a series of experiments were operated to optimize the determination conditions. The details of the optimizing experiments were proposed in the support information.

Under acidic conditions, the carbonyl group could react with 2,4-dinitrophenylhydrazine (DNPH) to generate 2, 4-dinitrophenylhydrazone, and the solution would turn into rufous which could be determined through spectrophotometry. Acetophenone was chosen as the standard organic matter containing the carbonyl group. Determination conditions were optimized by acetophenone, as well as the standard

curve. The absorption wavelength was 455 nm. The optimal dosage of 2, 4-DNPH was 1.0 mL. The reaction temperature was 50 ℃; The reaction time was 30 min. The chromogenic reaction time was 10 min.
